# PPAR-*δ* in Vascular Pathophysiology

**DOI:** 10.1155/2008/164163

**Published:** 2009-01-06

**Authors:** Nanping Wang

**Affiliations:** Laboratory of Vascular Molecular Biology and Bioengineering, Institute of Cardiovascular Science and Diabetes Center, Peking University Health Science Center, Beijing 100191, China

## Abstract

Peroxisome proliferator-activated receptors belong to the superfamily of ligand-dependent nuclear receptor transcription factors, which include three subtypes: PPAR-*α*, *β*/*δ*, and *γ*. PPAR-*δ*, play important roles in the regulation of cell growth and differentiation as well as tissue wound and repair. Emerging evidence has also demonstrated that PPAR-*δ* is implicated in lipids and glucose metabolism. Most recently, the direct effects of PPAR-*δ* on cardiovascular processes such as endothelial function and angiogenesis have also been investigated. Therefore, it is suggested that PPAR-*δ* may have critical roles in cardiovascular pathophysiology and is a potential target for therapeutic intervention of cardiovascular disorders such as atherosclerosis.

## 1. INTRODUCTION

Peroxisome proliferator-activated receptors (PPARs) are members of nuclear receptor/ligand-activated transcription factors superfamily. PPAR subfamily consists of 3 subtypes: PPAR-*α*, -*β*/*δ*, and -*γ*. PPARs form heterodimers with a retinoid X receptor (RXR) and bind to specific PPAR-responsive elements (PPREs) to regulate target gene expression. In the absence of specific ligands, the PPAR-RXR heterodimer forms repressive complex with corepressors and histone deacetylases. Upon ligand binding, the receptor undergoes conformational changes that cause the dissociation of repressors, the recruitment of coactivators, and the activation of gene transcription [1]. Recently, extensive studies have been performed to characterize the biological and pathophysiological roles of PPAR-*α* and -*γ*, which are pharmacological targets of the clinical interventions for dyslipidemia and type 2 diabetes, respectively. Fibrates class of lipid-lowering drugs such as fenofibrate and gemfibrozil are agonists for PPAR-*α*. Thiazolidinedione class of insulin sensitizers including troglitazone (Rezulin), rosiglitazone (Avandia), and pioglitazone (Actos) are specific ligands for PPAR-*γ* [2]. PPAR-*α*, primarily expressed in liver, muscles, heart, and kidney, plays a key role in fatty acid catabolism such as *β*-oxidation. PPAR-*γ* is highly expressed in fat, controls adipogenesis, and regulates insulin action. However, relatively little has been known with regards to the function of PPAR-*δ*, the only subtype of PPARs that is not a target of current drug. Newly developed synthetic ligands and genetically modified mouse models for PPAR-*δ* have rapidly advanced our understanding of the important roles of PPAR-*δ* in tissue development, repair, inflammation, and metabolism [3–7]. Most recently, the direct effects of PPAR-*δ* on cardiovascular processes such as endothelial function and angiogenesis have also been investigated. In this review, we will focus on the recent advancements regarding the roles of PPAR-*δ* in vascular pathophysiological processes.

## 2. MATERIALS AND METHODS

### 2.1. Gene and protein

PPAR-*δ*, also known as nuclear hormone receptor 1 (NUC1), PPAR-*β*, or NR1C2, was first cloned in 1992 [8, 9]. The PPAR-*δ* gene is mapped to human chromosome 6p21.2-p21.1 and has 11 exons, spanning 35 kilobase-pair [10]. Like other PPARs, PPAR-*δ* protein has a modular structure consisted of 5 regions: an N-terminal region (A/B), a DNA-binding domain (C), a flexible hinge region (D), ligand-binding domain (E), and a C-terminal region (F). X-ray crystallographic study revealed that PPAR-*δ* has an exceptionally large ligand binding pocket, which maybe related to the promiscuous accommodation of a large range of mostly amphipathic ligands [11, 12].

### 2.2. Endogenous and synthetic ligands

Several 14- to 18-carbon saturated fatty acids and 16- to 20-carbon polyunsaturated fatty acids can bind PPAR-*δ* [13–15]. Naturally occurring or synthetic eicosanoids such as prostaglandin A1 and carbaprostacyclin have been shown to bind and activate PPAR-*δ* [16]. Very low-density lipoprotein (VLDL) derived has also been demonstrated to activate the PPAR-*δ* target genes in a receptor-dependent manner [17]. Since these agonists activate PPAR-*δ* all with affinities at molar range, it raises a question as to whether these are bona fide physiological ligands for PPAR-*δ*. However, many of the above-mentioned agonists under the physiological or pathological conditions are either released by the vessels, such as PGI2, or being exposed to vascular endothelium, such as VLDL. It would be intriguing to examine whether PPAR-*δ* in the vessel wall is activated in vivo. In addition, it was recently shown that retinoic acid, a ligand for retinoic acid receptor, also can activate the PPAR-*δ* with nanomolar affinity without affecting the other two subtypes of PPARs [18]. This finding expanded our understanding of the mechanisms of PPAR-*δ* activation.

Recently, several synthetic ligands have been reported to selectively activate PPAR-*δ*. The PPAR-*δ* agonists reported to date were discovered using several strategies: GW501516 and GW-0742 (GlaxoSmithKline) were optimized from a library of hydrophobic carboxylates [19]; L165461 (Merck) was derived from an in silico approach [20]. These derivatives of phenoxyacetic acid are the highly selective PPAR-*δ* ligands with a nanomolar affinity and 1000-fold selectivity over PPAR-*α* and -*γ*. Other PPAR-*δ* agonists including KD3010 (Kalypsys) and MBX-8025 (Metabolex) are currently in clinical development. On the other hand, the development of these specific agonists has greatly aided the investigation in the biological functions of PPAR-*δ*. A selective antagonist for PPAR-*δ*, GSK0660, has also been recently demonstrated as it by itself exhibits inverse agonist activity and competes with agonist in a cellular context [21] (Table 1).

### 2.3. Effects on the vessel wall

Although nearly ubiquitously expressed with highest levels in placenta, skeletal muscles, and adipose tissue, PPAR*δ* is also expressed in the vascular cells including endothelial cells [22], smooth muscle cells, and macrophages. Particularly, a number of studies during the past 2 years have demonstrated that PPAR-*δ* plays direct roles in various basic vascular processes such as apoptosis, survival, angiogenesis, and inflammation.

### 2.4. Endothelial apoptosis

Vascular endothelium, when unperturbed, is considered to provide a relatively nonadhesive and nonthrombotic interface. This characteristic is likely essential to physiological homeostasis. However, endothelial cells (ECs) can undergo apoptosis in vitro in response to a variety of pathophysiological conditions including hypoxia, proinflammatory cytokines, bacterial endotoxins, and atherogenic risk factors such as homocysteine and lipoproteins [23, 24]. EC apoptosis has been implicated in numerous pathophysiological processes, such as angiogenesis, thrombosis, and atherosclerosis. On the other hand, ECs produce a plethora of bioactive molecules to maintain vascular homeostasis. Among those, prostacyclin (PGI2) protects ECs from apoptosis. Although PPAR-*δ* has been previously documented to protect against the hypertonicity-induced apoptosis in renal cells [25] and the growth factor deprivation- or anoikis-induced apoptosis in keratinocytes [6], its role in vascular cells has been recently demonstrated. Liou et al. showed that PGI2 protects ECs from H_2_O_2_-induced apoptosis via the action of PPAR-*δ*. By inducing the expression of its target gene 14-3-3alpha, PPAR-*δ* prevents Bad-triggered apoptosis [26]. Treatment with L165041 or overexpression of PPAR-*δ* also has a similar effect. In addition, small interfering RNA-mediated knockdown of PPAR-*δ* abrogated the antiapoptotic effect, suggesting that the antiapoptotic role of PPAR-*δ* appeared to be specific for and dependent on the endogenous PPAR-*δ* receptor. Interestingly, gene expression of 14-3-3epsilon was also induced by PPAR-*δ* through a PPRE-independent mechanism and an interaction between PPAR-*δ* and CCAAT/enhancer binding protein (C/EBP) [27].

### 2.5. Endothelial activation

When exposed to proinflammatory stimuli such as tumor necrosis factor (TNF) or lipopolysaccharide (LPS), normally quiescent endothelium undergoes a phenotypic change, which is characterized by induction of proinflammatory and procoagulant factors such as adhesion molecules and tissue factor. Such a phenotypic conversion, referred to as EC activation, is implicated in a number of proinflammatory diseases including atherosclerosis and thrombosis [28]. PPAR-*α* and -*γ* have been previously shown to suppress EC expression of proinflammatory adhesion molecules such as intercellular adhesion molecule-1 (ICAM-1), vascular adhesion molecule-1 (VCAM-1), E-selectin, monocyte chemoattactant protein-1 (MCP-1), and ensuing recruitment of leukocytes [22, 29–32]. However, there has also been evidence that suggests a proinflammatory role of PPAR-*α* or -*γ* [33, 34]. Recent studies suggested that PPAR-*δ* also plays a role in inflammatory processes and atherosclerosis. In macrophages, the PPAR-*δ* agonist GW0742 inhibited lipopolysaccharide (LPS)-induced expression of proinflammatory genes, such as cyclooxygenase (COX)-2 and inducible nitric oxide synthase (iNOS) [35]. GW0742 reduced atherosclerotic lesions and decreased the expression of MCP-1 and ICAM-1 in the aorta of LDLR^−/−^ mice [36, 37]. Given the beneficial effects of PPAR-*δ* agonists on lipid profiles, it is likely that PPAR-*δ* agonists may inhibit endothelial activation by improving dyslipidemia. A direct anti-inflammatory effect was also demonstrated. In EAhy926 cells, Rival et al. found that L-165041, at high a concentration up to 100 *μ*M, inhibited TNF-*α*-induced VCAM-1 and MCP-1 expressions [38]. In primary culture of human umbilical vein ECs (HUVECs), specific agonists GW0742 and GW501516 inhibited the TNF-*α*- or interleukin-1*β*-induced expression of adhesion molecules and the monocyte adhesion to ECs. PPAR-*δ* agonist induced the gene expression of antioxidative enzymes, such as superoxide dismutase-1, catalase, and thioredoxin, and it decreased reactive oxygen species production in ECs. Unexpectedly, the anti-inflammatory effect not only persisted but it also was further enhanced after the decrease of PPAR-*δ* expression by siRNA knockdown [39]. Given the evidence that the ligand binding caused the dissociation of the transcription repressor BCL-6 from PPAR-*δ* and the subsequent association of BCL-6 with the VCAM-1 promoter region, this seemingly paradoxical result could be plausibly interpreted with the previously proposed PPAR-*δ*/Bcl-6 interaction action mode: the synthetic ligand binds to PPAR-*δ* and recruits the coactivators to replace the corepressors such as Bcl-6. The released corepressors relocate to repress the transcription of proinflammatory genes such as VCAM-1 and E-selectin and thus contribute to the vascular protection.

Ghosh et al. recently showed that the metabolism of endocannabinoids by the endothelial COX-2 coupled to the prostacyclin synthase activates PPAR-*δ*, which negatively regulates the expression of tissue factor (TF), the primary initiator of blood coagulation [40]. As COX-2 inhibitors suppressed PPAR-*δ* activity and induced TF expression, these results may help explaining the prothrombotic adverse effects of the cox-2 inhibitors rofecoxib and valdecoxib [41].

### 2.6. Angiogenesis

Angiogenesis is referred to the formation of new capillaries from the existing blood vessels. Physiological angiogenesis is involved in wound healing and aerobic exercise, whereas pathological or therapeutic angiogenesis is implicated in cardiovascular diseases, diabetic complications, inflammatory diseases, and cancers [42]. Recent studies have also linked metabolic homeostasis to angiogenesis and further interrogate the potential effects of PPARs on the angiogenic process [43, 44]. An earlier study demonstrated that the PPAR-*δ* agonist GW501516 dose-dependently stimulates HUVEC proliferation with increased mRNA expression of vascular endothelial growth factor *α* and its receptor flt-1 [45]. Later, GW501516 was shown to promote endothelial tube formation on an extracellular matrigel, EC outgrowth in a murine aortic ring model, and increased angiogenesis in the implanted matrigel plug assay in vivo through a PPAR-*δ*- and VEGF-dependent manner [22]. Most recently, Gaudel et al. found that treatment with GW0742 or muscle-specific overexpression of PPAR-*δ* promoted angiogenesis in mouse skeletal muscle [46]. Besides, arising from sprouts on existing vessels, vessels also arise from endothelial progenitorcells (EPCs), a process referred to as vasculogenesis [47]. Culminating evidence further suggests that circulating EPCs is capable of stimulating angiogenesis [48]. A recent study showed that the proangiogenic effects of human EPCs are in part dependent on the biosynthesis and release of PGI_2_, and subsequent activation of PPAR-*δ* [48, 49]. Furthermore, functional genomic approach provided evidence that silencing of PPAR-*δ* in the tumor microenvironment impairs angiogenesis and tumor growth, identifying PPAR-*δ* as one of a few hub nodes in the angiogenic network [49, 50]. Up-to-date results have been largely consistent and pointed toward a proangiogenic activity of PPAR-*δ*. In corroboration with this, Müller-Brüsselbach et al. found that the growth of syngeneic PPAR-*δ* wild-type tumors was impaired in PPAR-*δ* ^−/−^ mice, concomitant with a reduced blood flow and hyperplastic vascular structures, suggesting that PPAR-*δ* maybe required in tumor ECs for the formation of functionally mature vessels [51]. Nevertheless, a full understanding of the specific roles of PPAR-*δ* in specific scenarios of angiogenesis will be imperative for a safe and rational therapeutic strategy.

### 2.7. Smooth muscle cells

PPAR-*δ* is expressed in SMCs and is induced in response to platelet-derived growth factor (PDGF) in SMCs, which involved the phosphatidylinositol 3-kinase/Akt signaling pathway. Initial study using overexpression showed that PPAR-*δ* increased SMC proliferation, indicating a proliferation-promoting effect in SMCs [52]. However, L-165041, a selective PPAR-*δ* agonist, inhibited SMC proliferation and migration via inhibition of the PDGF-induced expression of cyclin D1 and cyclin-dependent kinase (CDK) 4 and cell cycle progression [53]. In SMCs, GW501516 increased the expression of transforming growth factor-*β*1 (TGF-*β*1) and the effect seemed to depend on endogenous PPAR-*δ*. Subsequently, TGF-*β*1 was likely responsible for suppression of the IL1*β*-induced expression of MCP-1 and proliferation of SMCs [54]. In rats, administration of L-165041 decreased neointima formation in balloon-injured carotid arteries [53]. Thus, synthetic PPAR-*δ* agonists appear to have antiproliferative and anti-inflammatory properties in SMCs. This is consistent with previous reports that adenovirus-mediated gene transfer of prostacyclin synthase, which produces the endogenous PPAR-*δ* ligand PGI2, inhibited SMCs proliferation and intimal hyperplasia [55–57].

### 2.8. Macrophages

Macrophage infiltration in vessel wall is known to play important role in atherogenesis. PPAR-*δ* is expressed in macrophages. During past years, the role of PPAR-*δ* in macrophage biology has been extensively studied. However, existing results still remain controversial. Oliver Jr. et al. showed that GW501516 in a human monocytic cell line increased the expression of ATP-binding cassette A1 (ABCA1) and Apo AI-mediated cholesterol efflux [58]. However, Vosper et al. found that a different PPAR-*δ* agonist, compound F, increased lipid accumulation in both human primary macrophages and THP-1 cells. Compound F induced the expression of genes involved in lipid uptake and storage such as class A and B scavenger receptors (SRA and CD36) but repressed key genes involved in lipid metabolism and efflux such as Apo E and cholesterol 27-hydroxylase [59]. In mouse macrophages, neither genetic loss of PPAR-*δ* nor treatment with the PPAR-*δ* agonists GW501516 or GW0742 significantly influenced cholesterol efflux or accumulation [36, 60]. Beside the effects on lipid trafficking, PPAR*δ* agonists have a potent anti-inflammatory effect in macrophages. Welch et al. first demonstrated that, in mouse peritoneal macrophages, PPAR-*δ* agonist GW0742 inhibited LPS-induced expression of COX-2 and iNOS [32, 35]. Recently, Barish et al. found that GW501516 in mouse macrophages suppressed the gene induction of MCP-1, -3, -5 by IL-1, interferon-*γ* (IFN-*γ*), and phorbol ester. The agonist treatment also inhibited transendothelial migration of THP-1 cells [61]. The anti-inflammatory effects of the agonist was lost in the receptor-deficient macrophages [60]. However, in other cell types such as epithelial cells, eosinophils, neutrophils, and lymphocytes, the PPAR-*δ* agonist was ineffective in inhibiting inflammatory processes, indicating that the effect is cell-type-specific [62].

### 2.9. Atherosclerosis

To date, several studies have been reported regarding the roles of PPAR-*δ* in atherosclerosis in different mouse models with different approaches. Lee et al. transplanted PPAR-*δ*-null bone marrow progenitor cells into LDL receptor-null (LDLR^−/−^) mice. Unexpectedly, the adoptive transfer of PPAR-*δ*-null macrophages led to a less severe atherosclerosis, suggesting that endogenous PPAR-*δ* maybe proatherogenic. Although overexpression or deletion of PPAR-*δ* in macrophages suggested that PPAR-*δ* is proinflammatory, the agonist GW501516 decreased MCP-1, seemingly having an opposite effect [60]. To reconcile this contradiction, they postulated an unconventional ligand-dependent transcriptional mechanism, which switches PPAR-*δ* between a “proinflammatory” and “anti-inflammatory”: in the absence of ligand, PPAR-*δ* sequesters a transcriptional repressor of inflammatory responses such as Bcl-6, permitting induction of proinflammatory genes; in the presence of ligand, PPAR-*δ* releases the repressor, which is then free to exert its anti-inflammatory effects. Following this loss-of-function approach, two independent studies examined the effect of the PPAR-*δ* agonist GW0742 on atherogenesis in high fat- and cholesterol-fed LDLR^−/−^ mice and yielded divergent results. In the first study, Li et al. found that GW7842 decreased gene expression of proinflammatory cytokines and adhesion molecules within atherosclerotic lesions but failed to alter the progression of atherosclerosis after 14 weeks of treatment (5 mg^−1^ kg^−1^ day^−1^). In another one, Graham et al. used female LDLR^−/−^ mice fed with a diet that induced moderate levels of hypercholesterolemia and observed that GW0742 reduced the lesion size at a higher dose (60 mg^−1^ kg^−1^ day^−1^) after 10 weeks of treatment [37]. Discrepancy between these two studies may be caused by differences in the levels of hypercholesterolemia and different drug doses used. However, the anti-inflammatory effect was generally consistent in both studies, regardless the different effects on the lesion sizes. It is likely that the anti-inflammatory properties of the PPAR-*δ* agonists on the vessel wall per se are not sufficient to attenuate the progression of atherosclerotic lesions if it is not corroborated by an efficient improvement of metabolic abnormalities. This notion is supported by the data from recently published results. Most recently, GW501516, which has a potent lipid-modifying capacity, has also been demonstrated to have a clear antiatherosclerotic property in apoE^−/−^ mice. Barish et al. showed that GW501516 significantly reduced atherosclerotic lesions with an increase in HDL level and a reduced expression of chemokines in the aorta and in macrophages [61]. Furthermore, in a model of angiotensin II-accelerated atherosclerosis (LDLR^−/−^ mice), Takata et al. confirmed the atheroprotective effect of GW0742 [63]. After 4 weeks of treatment, GW0742 at both doses (1 and 10 mg^−1^ kg^−1^ day^−1^) significantly inhibited the Ang II induction of atherosclerosis without altering blood pressure. This beneficial effect was likely mediated via the potent anti-inflammatory property since GW0742 increased vascular expression of Bcl-6, the regulators of G protein-coupled signaling (RGS4 and 5) in the artery and suppressed Ang II-induced activation of p38 and ERK in macrophages. However, the metabolic effect of GW0742 may also have contributed to the atheroprotective outcome because GW0742 significantly reduced plasma levels of insulin, glucose, leptin, and decreased triglycerides [63]. Overall, studies in mouse models suggest that PPAR-*δ* may have an attractive therapeutic target for the treatment of atherosclerosis.

### 2.10. Cardiovascular risk factors

In addition to the direct effects on the vessel wall, PPAR-*δ* also has profound effects on various metabolic parameters associated with cardiovascular diseases such as obesity, dyslipidemia, and insulin resistance.

### 2.11. Obesity

PPAR-*δ* deficiency causes embryonic lethality due to a placental defect. Some surviving PPAR-*δ* null mice had reduced fat mass [3], indicating a role of PPAR-*δ* in adipogenesis. Transgenic mice specifically expressing VP16-PPAR-*δ*, a constitutively active receptor, in adipose tissue had a reduced body weight, fat mass, and lower levels of circulating free fatty acids and triglycerides [64]. These animals were less susceptible to high-fat diet-induced obesity. In contrast, PPAR-*δ* null mice were more prone to weight gain on a high-fat diet. Similarly, GW501516 ameliorated diet-induced obesity [65]. It has been known that PPAR-*δ* activates genes involved in fatty acid oxidation and energy dissipation, such as carnitine palmitoyltransferase 1 (CPT1), acyl-CoA oxidase (AOX), and long chain acyl-CoA dehydrogenase (LCAD), uncoupling proteins. In addition to the direct effects on obesity, PPAR-*δ* agonists may also have regulatory effects on adipokine profile. For example, administration of L-165041 in rats increased the expression of visfatin and adiponectin, which are known to improve insulin sensitivity and are vasoprotective, but decreased the production of resistin in visceral adipose tissue [66].

### 2.12. Dyslipidemia

Increased levels of LDL and triglycerides and decreased HDL in plasma are independent risk factors for atherosclerosis and associated with metabolic syndrome as well. Recent studies have demonstrated that activation of PPAR-*δ* may modify lipid profile in animal models as well as in human. Oliver Jr. et al. first reported that GW501516 significantly improved dyslipidemia in obese primates with an increase in HDL and a decrease in LDL cholesterol and triglycerides [58]. The beneficial effect of GW0742 and L-165041 on HDL level was also observed in obese and nonobese mice [67, 68]. In addition to enhancing fatty acid oxidation in muscles, PPAR-*δ* agonists upregulated expression of ABCA1 in several types of cells, which may lead to an increase in HDL and cholesterol reverse transport. In intestinal cells, it also inhibited gene expression of Niemann-Pick C1-like 1, the key molecule for cholesterol absorption [67]. However, the precise mechanisms underlying these lipid-modifying effects still remain to be elucidated. In a small number of healthy human volunteers, GW501516 has been reported to increase HDL cholesterol level and improved the triglycerides clearance [69].

### 2.13. Insulin resistance and glucose homeostasis

It has been previously known that, in obese primates, GW501516 declined fasting insulin level [58]. GW501516 treated *ob/ob* mice also showed a significantly improved glucose tolerance and with a lower postprandial levels of plasma glucose and insulin [65]. In cultured myotubes, PPAR-*δ* agonists were found to directly stimulate glucose uptake independent of insulin action. The agonist-stimulated glucose uptake in myotubes appeared to require AMP-activated protein kinase (AMPK) but not PPAR-*δ* [70, 71]. However, GW501516 had no acute effect on glucose transport in rat skeletal muscles [72]. Recently, Lee et al. showed that PPAR-*δ* ^−/−^ mice were glucose intolerant. Euglycemic hyperinsulinemic clamp experiments showed that GW501516 improved insulin sensitivity in multiple tissues including hepatic and peripheral tissues. The agonist suppresses hepatic glucose output and increases glucose disposal [73]. Gene array analysis suggested that PPAR-*δ* might ameliorate hyperglycemia by increasing glucose flux through the pentose phosphate pathway, which is known to enhance de novo fatty acid synthesis. Thus, it could be a concern whether PPAR-*δ* improves hyperglycemia at the cost of exacerbation of hepatosteatosis, a problem commonly associated with metabolic syndrome and diabetes. However, recent studies demonstrated that PPAR-*δ* also had a beneficial effect on hepatosteatosis. In a diet-induced mouse model of nonalcoholic steaohepatitis, GW501516 reduced triglycerides accumulation in the livers. In a most recent study, it was demonstrated that PPAR-*δ* suppressed hepatic lipogenesis via the induction of insulin-induced gene-1 (insig-1) and the inhibition of lipogenic sterol-regulatory element binding prortein-1 (SREBP-1) activation. In obese diabetic mice, hepatic overexpression of PPAR-*δ* ameliorated hepatosteatosis [74]. Since Insig-1 is a critical regulator of lipid homeostasis, identification of the insig-1 as a target gene of PPAR-*δ* may also facilitate our understanding of the profound effects of PPAR-*δ* activation on adipogenesis and lipid metabolism [75, 76]. Recently, in a double-blind and randomized study, PPAR-*δ* agonist (10 mg o.d. GW501516) was given to a small number of healthy overweight subjects. The results showed that treatment with GW501516 for 2 weeks significantly reduced liver fat content by 20% without increasing oxidative stress [77].

### 2.14. Gene polymorphisms

Skogsberg et al. initially described 4 polymorphisms: −409C/T (in the promoter region), +73C/T (exon 1), +255A/G (exon 3), and +294T/C (exon 4). The +294T/C polymorphism showed a significant association with a metabolic trait. The homozygotes for the C allele had a higher plasma LDL and a tendency toward higher risk of CHD compared with homozygous carriers of the T-allele [78, 79]. In addition, there is a highly significant association between the rare C allele and lower plasma HDL concentrations in the female patients with mixed hyperlipidemia. Associations were also found for the C-allele with coronary heart disease and body mass index (BMI) [80, 81]. Chen et al. also demonstrated that, in “lipoprotein and coronary atherosclerosis study” (LCAS) subjects, the PPAR-*δ* SNPs were strongly associated with the dyslipidemia, the responses to statin, and the atherosclerotic lesions [82]. Vänttinen et al. investigated the effects of the PPAR-*δ* gene SNPs on tissue glucose uptake and suggested that the SNPs regulate insulin sensitivity primarily in skeletal muscles [83]. The PPAR-*δ* SNPs were genotyped in type II diabetes subjects and normal control. Although no significant association was detected with the risk of type II diabetes, several SNPs were associated with fasting plasma glucose and BMI [84]. In addition, the association has been found between the PPAR-*δ* SNPs and metabolic syndrome, and the association was influenced by dietary fat intake [85].

## 3. CONCLUSIONS

**Figure 1:**
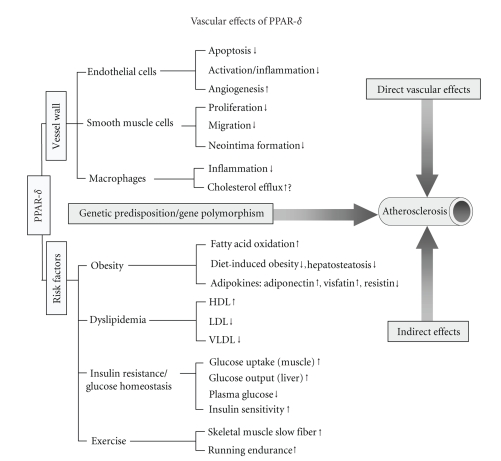
Activation of PPAR-*δ* may have profound effects on vascular homeostasis and coronary artery diseases. These include both the direct actions in the vessel wall and the indirect effects on multiple cardiovascular risk factors. PPAR-*δ* gene polymorphisms are also linked to cardiovascular diseases.

During the last few years, rapid progress has been made with regards to the roles of PPAR-*δ* in vascular biology. Emerging evidence supports the notion that activation of PPAR-*δ* may have profound effects on vascular homeostasis and coronary artery diseases. These include both the direct actions in the vessel wall and the beneficial effects on central metabolic pathways (Figure 1). Importantly, PPAR-*δ* agonists have an unprecedented role in raising HDL level in animals. In addition to a previously reported function of PPAR-*δ* in increasing oxidative muscle fibers and running endurance, a most recent study revealed that AMPK and PPAR-*δ* pathways have synergistic effects in terms of exercise-enhancing capacity [86]. The outcome of currently ongoing clinical trial is awaited to prove its clinical efficacy in the treatment of dyslipidemia. Despite the studies in rodent models point to a vascular-protective effect for PPAR-*δ* agonists, their efficacies in human coronary artery diseases remain to be clarified. With regards to the effects of PPAR-*δ* on tumor angiogenesis and the unsettled role in carcinogenesis, safety issues also call for attention [87, 88]. Clearly, further studies are warranted to explore the roles of PPAR-*δ* in cardiovascular pathophysiology and to exploit this lipid-sensing receptor as a therapeutic target for metabolic syndrome and its cardiovascular complications.

## Figures and Tables

**Table 1:** Ligands for PPAR-*δ*.

Ligands	Nature	Affinity	Clinical status
*Natural agonists*			
* * Linoleic acid	Dietary fatty acid	*μ*M
* * Oleic acid	Dietary fatty acid	*μ*M
* * Arachidonic acid	Dietary fatty acid	*μ*M
* * Eicosapentaenoic acid	Dietary fatty acid	*μ*M
* * Docosahexaenoic acid	Dietary fatty acid	*μ*M
* * Prostaglandin A1	Endogenous prostaglandin	*μ*M
*Synthetic agonists*			
* * Carbaprostacyclin	Synthetic stable PGI2 analogue	*μ*M
* * Iliprost	Prostacyclin analogue	*μ*M
* * Compound F	Phenoxyacetic acid derivative	nM
* * L165,041	Phenoxyacetic acid derivative	nM
* * GW501516	Phenoxyacetic acid derivative	nM
* * GW0742	Phenoxyacetic acid derivative	nM
* * KD2010	not disclosed	nM	Phase I
* * MBX-8025	not disclosed	nM	Phase II
*Synthetic dual agonists*			
* * Compound 23	Dual agonist for *γ* and *δ*	nM
*Synthetic antagonist*			
* * GSK0660	Antagonist for *δ*	nM
